# Selective growth inhibition of human malignant melanoma cells by syringic acid-derived proteasome inhibitors

**DOI:** 10.1186/1475-2867-13-82

**Published:** 2013-08-19

**Authors:** Khaled Y Orabi, Mohamed S Abaza, Khalid A El Sayed, Ahmed Y Elnagar, Rajaa Al-Attiyah, Radhika P Guleri

**Affiliations:** 1 Department of Pharmaceutical Chemistry, Faculty of Pharmacy, Health Sciences Center, Kuwait University, Safat 13110, Kuwait; 2 Department of Biological Sciences, Faculty of Sciences, Kuwait University, Safat 13110, Kuwait; 3 Department of Basic Pharmaceutical Sciences, College of Pharmacy, University of Louisiana at Monroe, Monroe LA 71201, USA; 4 Department of Microbiology, Faculty of Medicine, Kuwait University, Safat 13110, Kuwait; 5 Present address: Department of Pharmacology and Pharmaceutical Sciences, School of Pharmacy, University of Southern California, 1985 Zonal Ave, PSC 622, Los Angeles, CA 90033, USA

**Keywords:** Anti-mitogenic, Apoptosis, Molecular docking, Proteasome inhibitor, Rational design, Syringic acid, *Tamarixaucheriana*

## Abstract

**Background:**

It has been shown that proteasome inhibition leads to growth arrest in the G1 phase of the cell cycle and/or induction of apoptosis. However, it was found that some of these inhibitors do not induce apoptosis in several human normal cell lines. This selective activity makes proteasome inhibition a promising target for new generation of anticancer drugs. Clinical validation of the proteasome, as a therapeutic target in oncology, has been provided by the dipeptide boronic acid derivative; bortezomib. Bortezomib has proven to be effective as a single agent in multiple myeloma and some forms of non-Hodgkin’s lymphoma. Syringic acid (4-hydroxy-3,5-dimethoxybenzoic acid, **1**), a known phenolic acid, was isolated from the methanol extract of *Tamarix aucheriana* and was shown to possess proteasome inhibitory activity.

**Methods:**

Using Surflex-Dock program interfaced with SYBYL, the docking affinities of syringic acid and its proposed derivatives to 20*S* proteasome were studied. Several derivatives were virtually proposed, however, five derivatives: benzyl 4-hydroxy-3,5-dimethoxybenzoate (**2**), benzyl 4-(benzyloxy)-3,5-dimethoxybenzoate (**3**), 3*'*-methoxybenzyl 3,5-dimethoxy-4-(3*'*-methoxybenzyloxy)benzoate (**4**), 3*'*-methoxybenzyl 4-hydroxy-3,5-dimethoxybenzoate (**5**) and 3*'*,5*'*-dimethoxybenzyl 4-hydroxy-3,5-dimethoxybenzoate (**6**), were selected based on high docking scores, synthesized, and tested for their anti-mitogenic activity against human colorectal, breast and malignant melanoma cells as well as normal human fibroblast cells.

**Results:**

Derivatives **2**, **5**, and **6** showed selective dose-dependent anti-mitogenic effect against human malignant melanoma cell lines HTB66 and HTB68 with minimal cytotoxicity on colorectal and breast cancer cells as well as normal human fibroblast cells. Derivatives **2**, **5** and **6** significantly (p ≤ 0.0001) inhibited the various proteasomal chymotrypsin, PGPH, and trypsin like activities. They growth arrested the growth of HTB66 cells at G1 and G2-phases. They also arrested the growth of HTB68 cells at S- and G2-phase, respectively. Moreover, derivatives **2**, **5**, and **6** markedly induced apoptosis (≥ 90%) in both HTB66 and HTB68.

**Conclusions:**

Computer-derived syringic acid derivatives possess selective anti-mitogenic activity on human malignant melanoma cells that may be attributed to perturbation of cell cycle, induction of apoptosis and inhibition of various 26*S* proteasomal activities.

## Background

It is estimated that 10 million people worldwide are diagnosed with cancer and about 6.2 million die from the disease every year [1,2]. Tumour cells often have multiple alterations in their apoptotic mechanisms and/or signalling pathways that lead to increased levels of growth and proliferation [3,4]. Overriding these mutations stimulates the apoptotic signalling pathway, leading to tumour cell death, which is a significant area of focus in anticancer drug research.

Proteasomes are gaining escalating interest since they play a key role in cancer cell proliferation, inhibition of chemotherapy-induced apoptosis and drug resistant development. Proteasome is a multicatalytic protease complex that degrades most endogenous proteins, including misfolded or damaged proteins, to ensure normal cellular function. Proteasome degrades the majority of intracellular proteins, including p27^kip1^, p21, IkB-α, Bax, cyclins, metabolic enzymes, transcription factors and the tumour suppressor protein p53. In addition, several of its enzymatic activities (proteolytic, ATPase, de-ubiquitinating) demonstrate key roles in protein quality control, antigen processing, signal transduction, cell-cycle control, cell differentiation and apoptosis [5-7]. Therefore, proteasome is an attractive target for a combined chemoprevention/chemotherapeutic approaches and thus ideal for cancer therapy.

Recently, it has been shown that proteasome inhibition leads to growth arrest in the G1 phase of the cell cycle and/or induction of apoptosis [8,9]. However, it was found that some of these inhibitors do not induce apoptosis in several human normal cell lines [9-11]. This selective activity makes proteasome inhibition a promising target for new generation of anticancer drugs.

Clinical validation of the proteasome, as a therapeutic target in oncology, has been provided by the dipeptide boronic acid derivative; bortezomib [12]. Bortezomib has proven to be effective as a single agent in multiple myeloma [13] and some forms of non-Hodgkin’s lymphoma [14].

Despite the acceptable therapeutic index, patients treated with this drug in phases I and II clinical trials manifest several toxic side effects, including diarrhoea, fatigue, fluid retention, hypokalaemia, hyponatremia, thrombocytopenia, anaemia, anorexia, neutropenia and pyrexia [15,16]. These side effects justify the need to discover other safer proteasome inhibitors that are more readily available than synthetic drugs, e.g., natural products or nutritional compounds with pharmacophores similar to those of authentic proteasome inhibitors.

The pursuit for nontoxic natural proteasome inhibitors has been stimulated by the fact that several natural products, such as green tea polyphenols and the antibiotic lactacystin, have been shown to potently inhibit proteasome. One of the most promising drug candidates of this type is salinosporamide A, from the bacterium *Salinispora tropica*[17,18]. The introduction of salinosporamide into phase I clinical trials inspired the search for additional natural proteasome inhibitory scaffolds. Over the past two decades, only one FDA-approved drug (sunitinib for renal carcinoma in 2005) was discovered based on high-throughput screening of combinatorial chemistry libraries [19,20]. Natural product-based drugs (parent compounds, analogues, and mimics) are still the major new entities source among the FDA-approved drugs (57.7% of all drugs) [21,22].

TMC-95A, B, C and D, cyclic polypeptides isolated from *Apiospora montagnei*, were shown to reduce trypsin-like and peptidylglutamyl-peptide hydrolysing activity of the proteasomal 20S core particle at a nonmolar range. This activity data is indicative of a highly selective inhibitor for the 20S proteasome [21,22].

**Figure 1:**
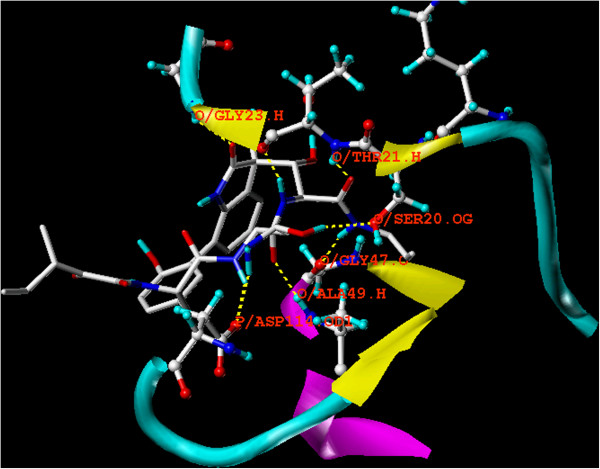
**Overlay docking alignment of TMC-95A.** Compound TMC-95A docked into in the active site of 20S yeast proteasome (PDB code: 1JD2). Selected structure is of 3D resolution 3.0 Å.

Since these cyclic polypeptides are not related to any previously reported proteasome inhibitor, their proteasome binding mode was determined through crystallographic analysis. Crystal structure of TMC-95A-proteasome complex indicates a non-covalent linkage to the active β-subunits, Figure 1. This binding mode does not modify these β-subunits’ *N*-terminal threonine residue, in contrast to all previous structurally analysed proteasome-inhibitor complexes.

The natural product syringic acid, known chemically as 4-hydroxy-3,5-dimethoxybenzoic acid, was recently isolated from the methanol extract of *Tamarix aucheriana*. Additionally, the preliminary results showed that this phenolic acid possesses potent anti-proliferative activity against human colorectal and breast cancer cells.

Computer-assisted drug design technique plays an important role in drug design and discovery, as well as in preliminary prediction of mechanisms *via in silico* exploration of possible binding sites of the target macromolecule in a non-covalent fashion [23,24].

This report accounts on attempts made to optimize syringic acid proteasome inhibitory activity via rational design of some active semisynthetic derivatives. Several virtual semisynthetic syringic acid derivatives were designed and docked at the active site of 20*S* proteasome core particle. Syringic acid derivatives with high docking scores were selected, synthesized and their proteasome inhibitory activities were studied *in vitro*.

## Results and discussion

### Chemistry

**Scheme 1:**
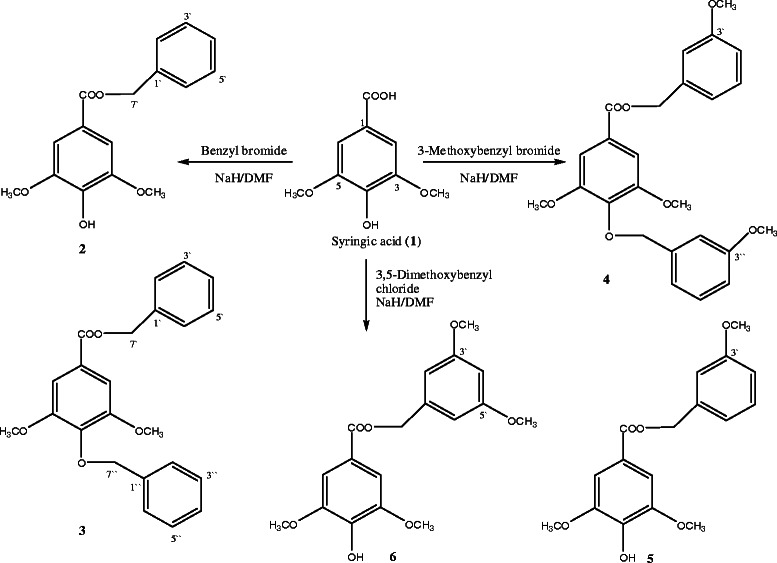
**Semisynthetic routes for syringic acid derivatives 2–6.**

Eighteen virtual aromatic, heteroaromatic, aliphatic, and olefinic esters, thioesters, carbamates, and ethers of syringic acid were proposed to explore the electronic space around the carboxy and free phenol groups. These structures were docked at the active site of available crystal structures of 20*S* proteasome (PDB 1R0P and 1JD2). Of these structures, syringic acid semisynthetic derivatives **2**–**6**, assessed in this study, were selected for chemical synthesis. This selection was based upon two criteria; the high docking score and the feasibility of chemical synthesis. The route used for the semisynthesis of these derivatives is shown in Scheme 1. These derivatives were synthesized directly, in good yields, by refluxing equimolar quantities of syringic acid with benzyl halides in *N,N*-dimethyl formamide, followed by reaction work up, extraction and chromatographic purification. The identity of the pure derivatives was confirmed based on their spectral data.

### Biological activity

#### Dose-dependent anti-mitogenic effect of syringic acid derivatives on human cancer cells and normal human fibroblast

##### Derivative 2

The dose-dependent antimitogenic activity of **2** (0.1 – 2 mg /mL) towards a panel of human breast (HTB26, HTB132), malignant melanoma (HTB66) and colorectal (CCL233, CCL235) cancer cell lines as well as normal human fibroblast (CRL1554) were tested after 144 h of treatment.

**Figure 2:**
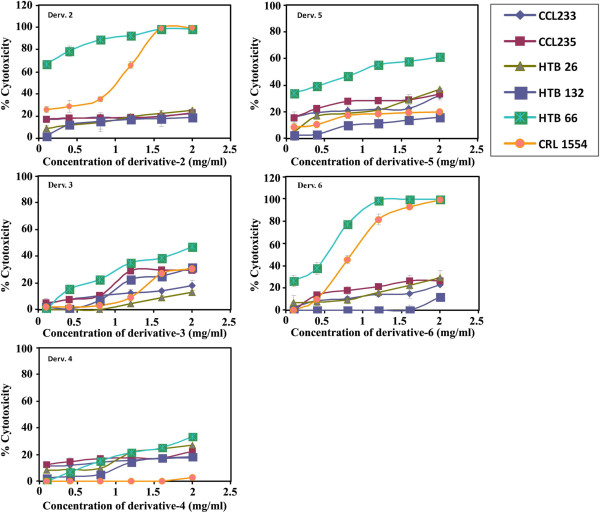
**Dose-dependent anti-mitogenic effect of derivatives 2–6 on different human cancer cell lines.** Human cancer cell lines (CCL233, CCL235, HTB26, HTB132, HTB 66, HTB 68) and normal fibroblast (CRL1554) were plated (27x10^3^ cells/well) into 96 well plates and incubated at 37°C in a CO_2_ and non-CO_2_ incubators. Cells were treated with different concentrations of derivatives (0.1-2 mg/mL) for 144 h. The cell growth was measured by MTT assay.

**Figure 3:**
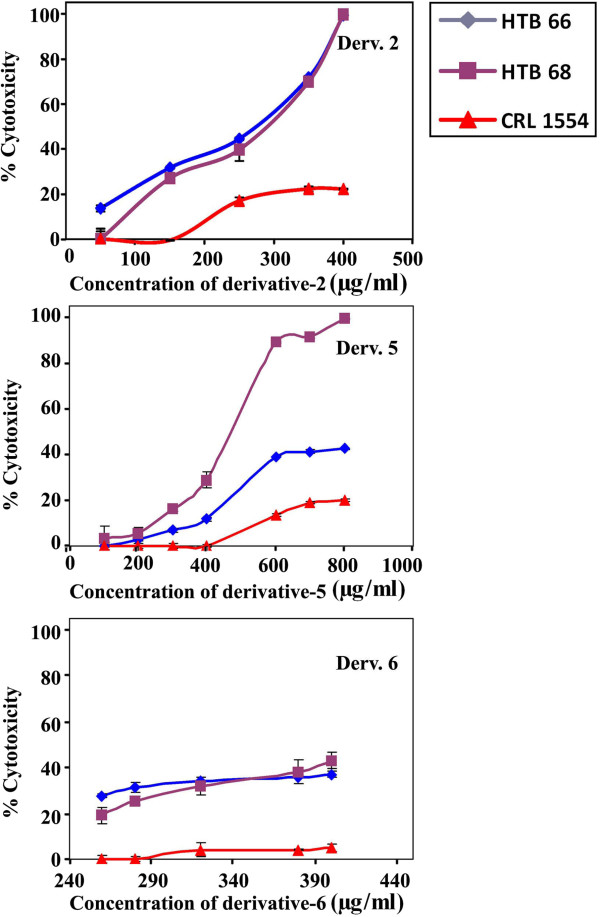
**Dose-dependent anti-mitogenic effect of derivatives 2, 5 and 6 on human melanoma cancer cell lines.** Human melanoma cell lines (HTB66 and HTB68) and normal fibroblast (CRL1554) were plated (27x10^3^ cells/well) into 96 well plates and incubated at 37°C in a CO_2_ incubator. Cells were treated with 2 (50–400 μg/mL), 5 (100–800 μg/mL), and 6 (260–400 μg/mL) for 24 h. The cell growth was measured by MTT assay.

All tested cancer cell lines, except melanoma, showed a maximum growth inhibition of about 20% (Figure 2). Melanoma cells exhibited a dose-dependent growth inhibition. However, normal human fibroblast showed a marked growth inhibition at a concentration higher than 1.0 mg/mL (Figure 2). The anti-mitogenic activity of **2** towards malignant melanoma was retested using lower concentrations of (50, 150, 259, 350 and 400 μg/mL) and less exposure time, 24 h. Under these conditions, **2**, at 50–400 μg/mL, exerted a marked significant growth inhibition on human malignant melanoma cells HTB66 (% mean of cytotoxicity = 52.2 ± 8, IC_50_ = 266.7 μg/mL, *p* ≤ 0.0001) and HTB68 (% mean of cytotoxicity = 47.2 ± 9, IC_50_ = 280 μg/mL, *p* ≤ 0.002) compared to the effect of **2** on normal human fibroblast CRL1554 (% mean of cytotoxicity = 12.7 ± 2.9, Figure 3).

These results are consistent with previous studies on the growth inhibitory effect of other plant phenolic acids against different types of cancer cells [25,26].

##### Derivatives 3 and 4

These derivatives were tested for their anti-mitogenic activities, at different concentrations and 144 h exposure time towards human colorectal, breast, malignant melanoma cancer cell lines and normal human fibroblast. Derivatives **3** and **4** showed a maximum growth inhibition, between 25-40%, on human melanoma (HTB66, 40%), colorectal (CCL235, 30%) and breast (HTB132, 25%) cancer cell lines. Meanwhile, colorectal (CCL233) and breast (HTB26) cancer cell lines as well as normal human fibroblast CRL1554 showed a maximum growth inhibition of 10%. These results showed that derivatives **3** and **4** possess low anti-mitogenic activities (Figure 2). Derivatives **3** and **4** were not further investigated due to their low antimitogenic activities and low synthetic yield.

##### Derivatives 5 and 6

Dose-dependent anti-proliferative effects of derivatives **5** and **6** towards human colorectal, breast, malignant melanoma cancer cell lines and normal human fibroblast were tested after 144 h of treatment. The inhibition study indicated that derivative **5** exerted a higher growth inhibition of malignant melanoma (> 60%) compared to other cancer cell lines (≤ 30%) and normal fibroblast (20%) that were slightly affected (Figure 3). Lower concentrations of derivative **5** (100–800 μg/mL) were retested against human malignant melanoma and normal fibroblast. It showed a higher growth inhibitory effect on malignant melanoma HTB66 (% mean of cytotoxicity = 21 ± 4, IC_40_ = 600 μg/mL, *p* ≤ 0.115) and HTB68 (% mean of cytotoxicity = 49 ± 9, IC_40_ = 440 μg/mL, *p* ≤ 0.001) compared to the normal fibroblast (% mean of cytotoxicity = 7.8 ± 2) (Figure 3). On the other hand, **6** had a maximum growth inhibitory effect of 20% on the tested cancer cell lines except for human malignant melanoma (HTB66) cells that were markedly inhibited in a dose-dependent manner. However, normal fibroblast cells were also greatly affected. So, lower concentrations of derivative **6** (260, 280, 320, 380 and 400 μg/mL) were retested after 24 h of treatment. Derivative **6** (260–400 μg/mL) produced a greater growth inhibition of HTB66 (% mean of cytotoxicity = 33.2 ± 1, IC_40_ = 398.7 μg/mL, *p* ≤ 0.0001) and HTB68 (% mean of cytotoxicity = 31.5 ± 2.7, IC_40_ = 380.6 μg/mL, *p* ≤ 0.0001) compared to the normal human fibroblast CRL1554 (% mean of cytotoxicity = 3.5 ± 0.9, Figure 3).

These results are in agreement with those reported for other phenolic acids in different types of cancers [27-30].

#### Inhibition of proteasomal activities in human malignant melanoma cell extracts by derivatives 2, 5 and 6

The potential of derivatives **2**, **5** and **6** to inhibit the proteasomal activities in human malignant melanoma cell extracts were evaluated by measuring the various proteasomal proteolytic activities, chymotrypsin-like, trypsin-like and PGPH, after treatment with derivative **2** (2 mg/mL), derivative **5** (1.3 mg/mL) or derivative **6** (1.75 mg/mL).

**Figure 4:**
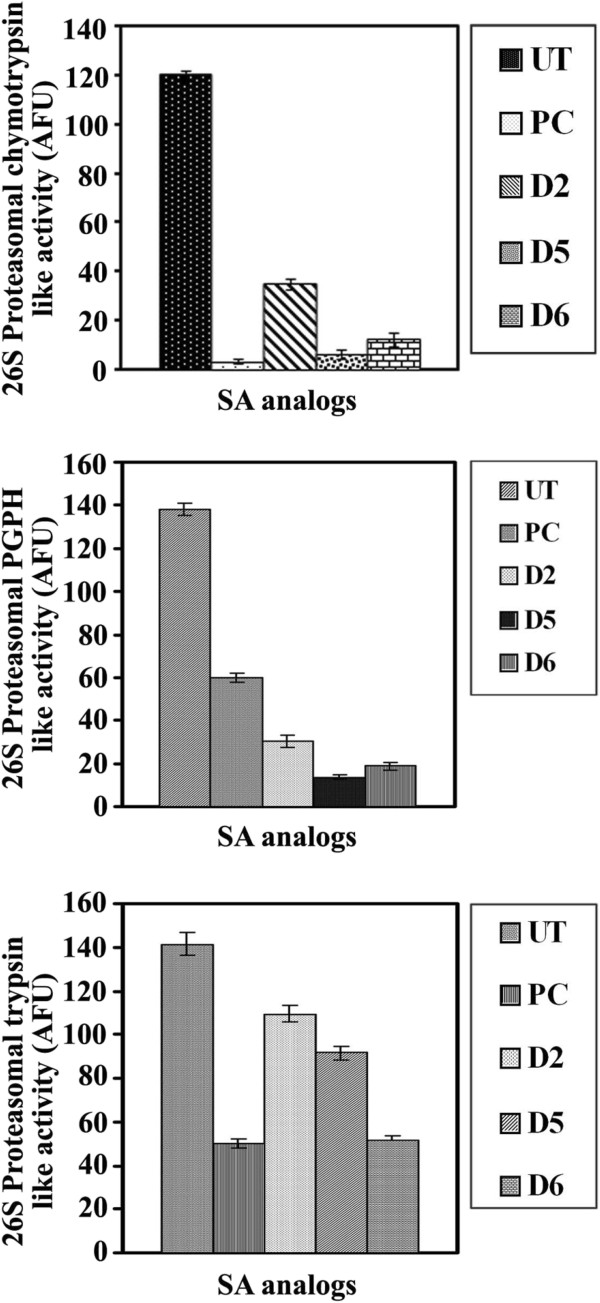
**Inhibition of 26S proteasomal activities of human malignant melanoma cells treated with derivatives 2, 5 and 6.** Human melanoma cells HTB68 were plated (1 × 10^6^ cells/well) in 12 well plates and incubated 24 h at 37°C in CO_2_ incubator. Cells were then treated with 2 (1.5 mg/mL), 5 (2.6 mg/mL) and 6 (1.75 mg/mL) or epoxomicin (50 ng/mL) as a positive control for 24 h. Cells were harvested and cytosolic fractions were prepared using nuclear/cytosol fractionation assay kit (Bio-Vision incorporated). The chymotrypsin-like activity, PGPH activity and trypsin-like activity of 26S proteasome was monitored using fluorogenic substrates in fluorometer with an excitation filter of 360 nm and emission filter of 460 nm.

All the tested derivatives produced a significant (*p* ≤ 0.0001) inhibition of proteasomal chymotrypsin-like activity. Moreover, derivatives **2**, **5** and **6** exhibited a significant (*p* ≤ 0.0001) inhibition of proteasomal PGPH-like activity. Furthermore, derivatives **2**, **5** and **6** exerted a significant reduction (*p* ≤ 0.0001) of proteasomal trypsin-like activity (Figure 4) compared to untreated malignant melanoma. Derivatives **3** and **4** were not tested because of their low anti-mitogenic activities and low synthetic yields, as well.

These results are consistent with those reported for other natural products, that exhibited anti-proteasomal activity in various human cancers, such as epigallocatechin gallate (EGCG) [31-33], gallic acid [25], quercetin [26], apigenin, a mixture of quercetin and myricetin [34], curcumin [35-37], genistein [38] and EGCG analogues [39,40].

How derivatives **2, 5** and **6** disturb the cellular proteasome function yet to be discovered. They could inhibit the proteasome function directly by blocking the 20*S* proteasome core cavity, or indirectly either by inhibiting the ubiquitin isopeptidase activity, or through the generation of oxidative stress. Inhibition of isopeptidase activity probably leads to the accumulation of ubiquitin-protein conjugate and polyubiquitin because of the lack of ubiqui-tin recycling process. Excessive accumulation of ubiquitin-protein conjugates could conceivably create proteasomal dysfunction. Derivatives **2, 5** and **6** may also induce proteasomal malfunction through the generation of oxidative stress. Oxidative stress is known to inhibit the proteasome function [41,42]. Impairment of proteasome function by derivatives **2, 5** and **6** warrants further investigation.

#### Effect of syringic acid derivatives on human malignant melanoma cell cycle

**Figure 5:**
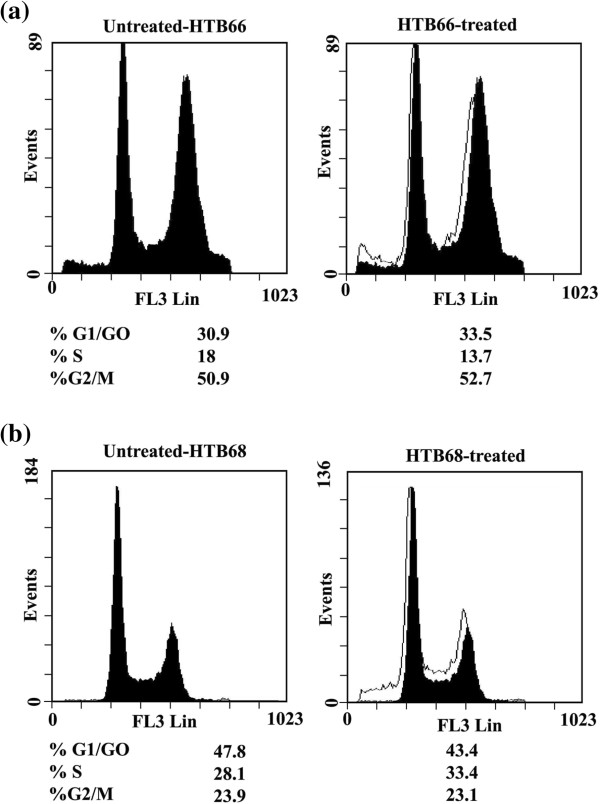
**Flow cytometric analysis of cell cycle distribution of human melanoma cells treated with 2.** Human melanoma cell lines HTB 66 **(a)** and 68 **(b)** were treated with 2 (1.3 mg/mL), for 24 h, starting 18 h after seeding the cell in culture. At least 3 samples were analysed and 20,000 events were scored for each sample. The vertical axis represents the relative number of events and the horizontal axis represents the fluorescence intensity.

**Figure 6:**
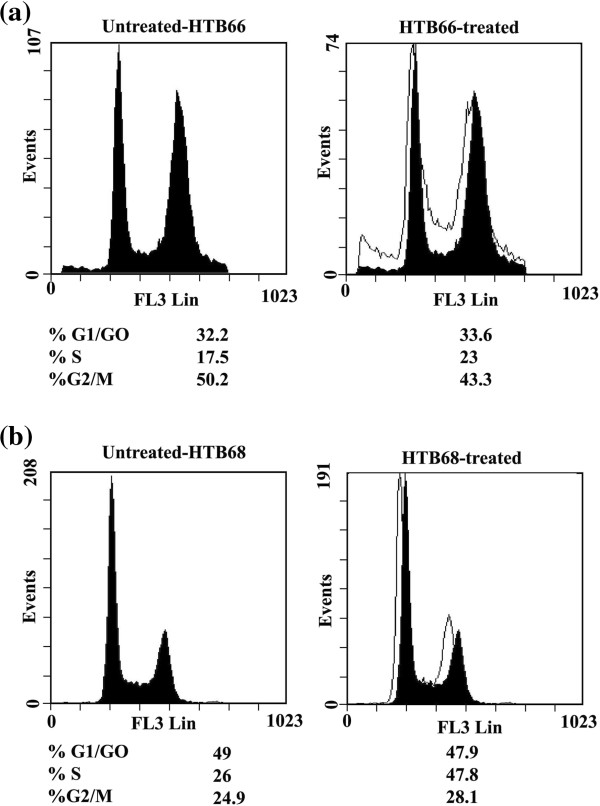
**Flow cytometric analysis of cell cycle distribution of human melanoma cancer cells treated with 5.** The human melanoma cancer cell lines HTB66 **(a)** and 68 **(b)** were treated with 5 (1.9 mg/mL), for 24 h, starting 24 h after seeding the cell in culture. At least 3 samples were analysed and 20,000 events were scored for each sample. The vertical axis represents the relative number of events and the horizontal axis represents the fluorescence intensity.

Treatment of human malignant melanoma cell line HTB66 with 1.3 mg/mL of **2** for 24 h arrested the growth of HTB66 cells at G_1_-phase (33.5% *vs.* 30.9% for UT) and G_2_-phase (52.7% *vs*. 50.9%; untreated, UT) with corresponding decrease in HTB66 cells in S-phase (13.7% *vs.* 18% for UT) (Figure 5a). On the other hand, derivative **2** arrested the growth of human malignant melanoma HTB-68 at S-phase (33.4% *vs*. 28.1% for UT) with corresponding decrease in HTB-68 cells in G_1_-phase (43.4% *vs*. 47.8% for UT) and G_2_-phase (23.1% *vs*. 23.9 for UT) (Figure 5b). Moreover, treatment of malignant melanoma cell line HTB66 with **5** (1.9 mg/mL) for 24 h arrested HTB66 growth at S-phase (23% *vs*. 17.5 for UT) and G1-phase (33.6% *vs*. 32.2 for UT) with corresponding decrease in HTB66 cells at G_2_-phase (43.3% *vs*. 50.2% for UT) (Figure 6a). On the other hand, **5** arrested HTB68 growth at G_2_-phase (28.1% *vs*. 24.9% for UT) with corresponding decrease in HTB68 cells at G_1_-phase (47.9% vs. 49% for UT) and S-phase (23.8% *vs*. 26% for UT, Figure 6b).

#### Induction of apoptosis in human malignant melanoma treated with derivatives 2 and 5

**Figure 7:**
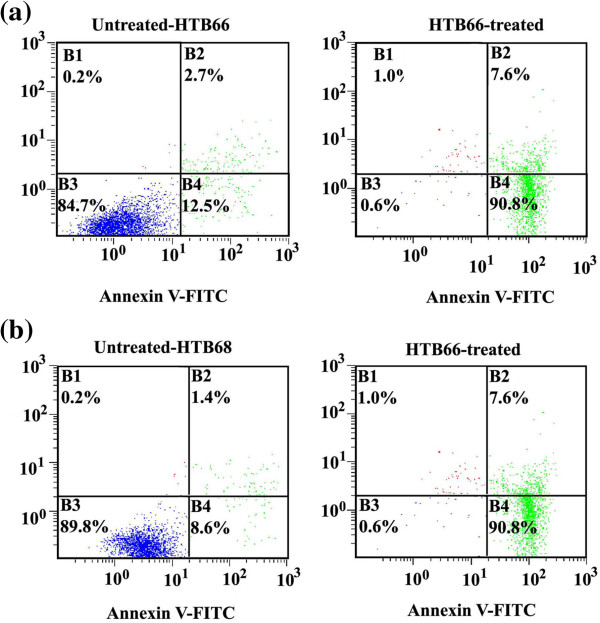
**Assessment of apoptosis in human malignant melanoma cells by annexin V-FITC and propidium iodide staining.** Untreated and derivative 2-treated (1.3 mg/mL, for 24 h) human malignant melanoma cell lines HTB66 **(a)** and HTB68 **(b)** were stained with annexin V-FITC and propidium iodide, then analysed by flow cytometry. B4: percentage of early apoptotic cells, B2: percentage of late apoptotic cells, B1: percentage of necrotic cells and B3: living cells.

The induction of apoptosis has been recognized as an effective tool in the therapeutic treatment of many tumours. In the present study, treatment of human malignant melanoma cell lines HTB66 and HTB68 with 1.3 mg/mL of **2** for 24 h, markedly induced apoptosis in HTB66 (% early apoptosis = 90.8% and % late apoptosis = 7.6% *vs*. 12.5% and 2.7% for early and late apoptosis, respectively, in UT) (Figure 7a) and HTB68 (% early apoptosis = 90.8% and % late apoptosis = 7.6% *vs*. 8.6% and 1.4% for early and late apoptosis, respectively, in UT, Figure 7b). Similar marked induction of apoptosis was noticed when malignant melanoma cell lines were treated for 24 h with 1.9 mg/mL of **5**. Derivatives **2** and **5**-induced apoptosis is mediated through the impairment of the ubiquitin-proteasome system.

When proteasome inhibitors prevent the proteasome from activating NFκB, factors of angiogenesis, survival, and growth are down regulated while apoptosis is up regulated in multiple cell lines [43,44]. This effect is also noticed in chemotherapy-resistant cells, additionally due to disruption of proteasomal regulation of caspases and Bcl2. Further, proteasome inhibition enhances the levels of p21 and p27 [45]. Such enhancement inhibits CDKs and consequently arrests cell cycle and halting the growth of cancer cells. The inhibition of the proteolytic function of the 26*S* proteasome has also been shown to impair the development of new blood vessels from endothelial cells or angiogenesis that is a vital factor for tumour growth and metastasis [46]. Disruption of angiogenesis by proteasome inhibition also occurs by decreasing microvessel density and the expression of vascular endothelial growth factor (VEGF) [46]. Thus, the proteasomal inhibition impairs angiogenesis as well as disturbs cellular homeostasis, hence leading to an antitumor activity. Overall, the inhibition of the proteolytic function of the 26*S* proteasome induces apoptosis and cell cycle arrest, and represses angiogenesis as well as metastasis. In fact, apoptosis and other antitumor effects have been observed in various cancer cell lines and xenograft models including lymphoma, leukaemia, melanoma, pancreatic, prostate, head and neck, breast, and lung cancers [47,48]. Further, cancer cells are more sensitive to the cytotoxic effects of the proteasome inhibition as compared to the normal cells [49]. Also, cessation of all proteasomal function is not required to achieve antitumor effects [50]. Together, these studies have implicated the proteasome inhibition as an attractive way of treating cancer cells. Several proteasome inhibitors have shown significantly improved antitumor activities when combined with other drugs such as HDAC inhibitors, Akt inhibitors, DNA damaging agent, Hsp90 inhibitor, and lenalidomide. In summary, proteasome inhibitor alone or in combination with other therapies have shown very promising results to treat cancer patients in the clinic more effectively.

The selectivity of the antitumor spectrum activity of syringic acid derivatives towards human malignant melanoma cells may be associated with several mechanisms which may be speculated to include disruption of cell adhesion- and cytokine-dependent survival pathways, e.g., NFκB signalling pathway, inhibition of angiogenesis, activation of a misfolded protein stress response (or ER stress), up regulation of proapoptotic or down regulation of antiapoptotic genes. DNA microarray analysis of the expression of genes controlling these regulatory mechanisms in melanoma cells-treated with syringic acid derivatives will clarify the selectivity of the antitumor activity of these derivatives against human malignant melanoma cells.

### Molecular modelling studies

Bortezomib is the best described proteasome inhibitor and the first to be clinically tested in humans, especially against multiple myeloma and non-Hodgkin’s lymphoma. Therefore, bortezomib was selected as a reference standard in this study. Bortezomib acts by binding β5i and β1i proteasome subunits [7].

In its bound conformation, bortezomib adopts an antiparallel β-sheet conformation filling the gap between strands S2 and S4. These β-sheets are stabilized by direct hydrogen bonds between the conserved residues (Gly47N, Thr21N, Thr21O, and Ala49O) of the β-type subunits and main chain atoms of the drug [21]. Both Thr21O and Ala49N, conserved in all proteolytically active centres, are essential for β-sheet formation. Their respective carbonyl oxygen and nitrogen atoms tightly interact with bortezomib’s pyrazine-2-carboxyl-phenylalanyl peptide backbone. The binding mode and conformation was found to be uniform in all proteolytically active sites [21].

Docking of syringic acid derivatives showed that the binding modes of energy-minimized derivatives are similar to bortezomib bound conformation to crystal structure of the eukaryotic yeast 20*S* proteasome which was obtained from the Protein Database (PDB code: 2 F16).

**Figure 8:**
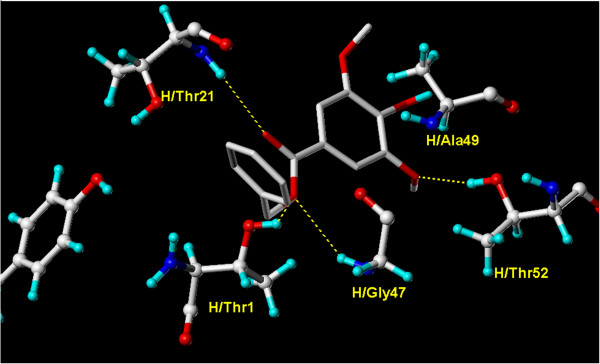
**Overlay docking alignment of derivative 2.** Derivative 2 docked in the active site of 20S yeast proteasome (PDB code: 2 F16). Selected structure is of 3D resolution 2.8 Å.

**2** demonstrated a good binding score presented in total score as compared to bortezomib (Table 1). The carboxyl moiety of the ester link of **2** formed three hydrogen bonds with H/Thr1, H/Gly47 and H/Thr21. Furthermore, one hydrogen bond was formed between the methoxyl group and H/Thr52 as shown in Figure 8.

**Table 1:** Virtual binding scores of syringic acid analogues and bortezomib

**Compound**	**Total score**	**Crash**	**Polar**
Bortezomib	8.50	−2.13	2.26
**4**	6.59	−1.76	1.93
**3**	6.58	−1.43	3.11
**6**	6.32	−1.31	2.07
**5**	6.06	−1.18	3.13
**2**	5.43	−0.86	3.05
Syringic acid	3.83	−1.52	1.45

Using SYBYL-X Surflex-Dock and the eukaryotic yeast 20S proteasome crystal structure (PDB code: 2 F16). Total score was expressed in –log (K_d_) units to represent binding affinities. Crash is the degree of inappropriate penetration by the ligand into the protein and of interpenetration between ligand atoms that are separated by rotatable bonds. Polar score is the contribution of the polar non-hydrogen bonding interactions to the total score. The polar score may be useful for excluding docking results that make no hydrogen bonds.

On the other hand, derivatives **3** and **4** showed the best binding score, presented in total score, when compared to other derivatives (Table 1). These results were in contrary to what one would expect for *in vitro* activities, where **3** and **4** were shown to be the least active derivatives. One reason for these unexpected low biological activities might be their poor water-solubility when compared to the other ones. In derivatives **3** and **4**, the phenolic and carboxylic hydroxyl groups were etherified and esterified, respectively. This dramatically reduced their polarity, expected water-solubility, and hence, limited their available critical concentrations needed for bioactivities.

**Figure 9:**
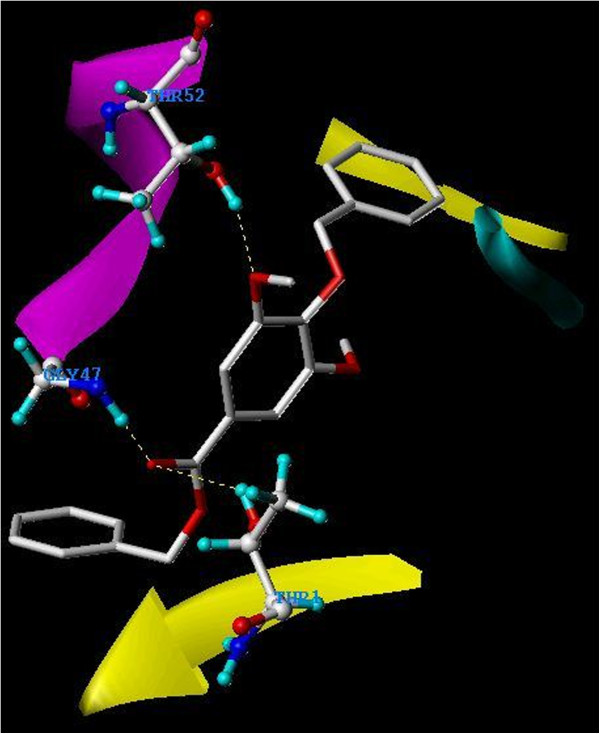
**Overlay docking alignment of derivative 3.** Derivative **3** docked in the active site of 20S yeast proteasome (PDB code: 2 F16). Selected structure is of 3D resolution 2.8 Å.

**Figure 10:**
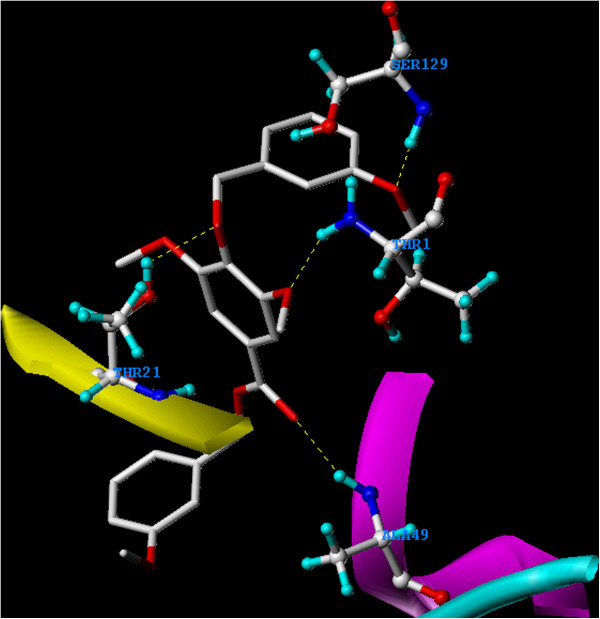
**Overlay docking alignment of derivative 4.** Derivative 4 docked in the active site of 20S yeast proteasome (PDB code: 2 F16). Selected structure is of 3D resolution 2.8 Å.

The carboxyl moiety of the ester linkage of **3** formed two hydrogen bonds with H/Gly47 and H/Thr1. Another hydrogen bond was present between one of the methoxyl groups of syringic acid and H/Thr52, as shown in Figure 9. On the other hand, the carboxyl moiety of the ester linkage of **4** formed a hydrogen bond with H/Ala49. Another hydrogen bond was formed between one of the methoxyl groups of syringic acid and H/Thr1, while a third hydrogen bond was formed between the ether linkage and H/Thr21. Additional hydrogen bond was also seen between the *m*-methoxyl group of the newly added benzyl ether moiety and H/Ser129 (Figure 10).

**Figure 11:**
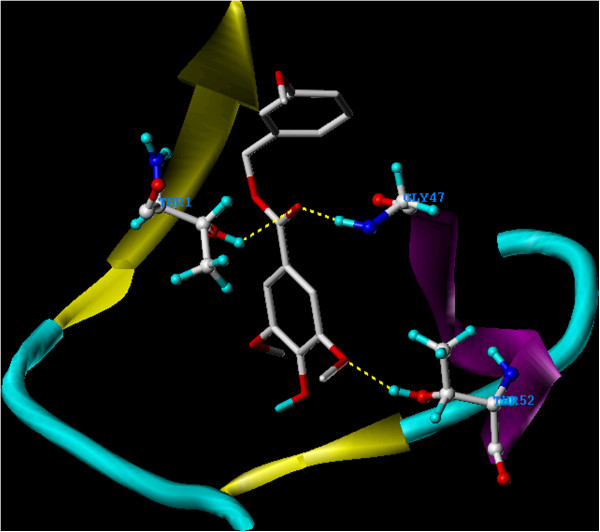
**Overlay docking alignment of derivative 5.** Derivative 5 docked in the active site of 20S yeast proteasome (PDB code: 2 F16). Selected structure is of 3D resolution 2.8 Å.

**Figure 12:**
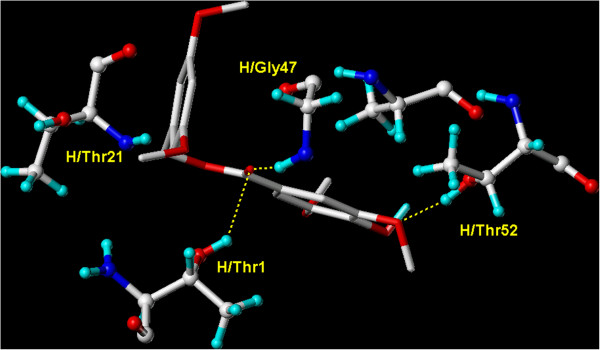
**Overlay docking alignment of derivative 6.** Derivative 6 docked in the active site of 20S yeast proteasome (PDB code: 2 F16). Selected structure is of 3D resolution 2.8 Å.

Moreover, **5** showed a slightly higher binding score than **2** (Table 1), however, it demonstrated a similar binding conformation to **2** (Figure 11). Finally, **6** showed a comparable binding score and a similar docking conformation to **3** (Figure 12).

## Conclusions

Out of eighteen syringic acid derivatives virtually proposed, only five derivatives; benzyl 4-hydroxy-3,5-dimethoxybenzoate (**2**), benzyl 4-(benzyloxy)-3,5-dimethoxybenzoate (**3**), 3′-methoxybenzyl 3,5-dimethoxy-4-(3′-methoxybenzyloxy) benzoate (**4**), 3′-methoxybenzyl 4-hydroxy-3,5-dimethoxybenzoate (**5**) and 3′,5′-dimethoxybenzyl 4-hydroxy-3,5-dimethoxy-benzoate (**6**), showed high binding affinity and, therefore, were chemically synthesized.

Syringic acid derivatives **2, 5** and **6** were shown to inhibit human malignant cell growth, and proteasome activity, and apoptosis inducers. Proteasome inhibitors are considered promising anticancer agents. Therefore, syringic acid derivatives **2**, **5** and **6**, with their safe profile on normal human fibroblasts, have enormous potential for future use for the prevention and control of human malignant melanoma. The intimate coupling of multicomponent computer modelling with natural products-based prospecting, in bidirectional fashion and the use of *in silico* and *in vitro* tools for efficacy and selectivity optimization, provide guidance and perfect examples of rational drug discovery and design approaches.

## Methods

### Chemistry

The IR spectra were recorded as neat solids using an FT/IR-4100 JASCO spectrophotometer. The ^1^H and ^13^C NMR were obtained on a Bruker Avance II-600 spectrometer operating at 600 and 125 MHz, respectively. Both ^1^H and ^13^C NMR spectra were recorded in CDCl_3_, and the chemical shift values were expressed in *δ* (ppm) relative to the internal standard TMS. For the ^13^C NMR spectra, the number of attached protons was determined by DEPT 135°. 2D NMR data were obtained using the standard pulse sequence of the Bruker Avance II-600 for COSY, HSQC, and HMBC. Mass Spectroscopy was carried out using a Bruker Bioapex FTMS with Electrospray Ionization Spectrometer. Thin layer chromatography was performed on pre-coated (0.25 mm) silica gel GF_254_ plates (E. Merck, Germany) and compounds were visualized *via* exposure to 254 nm UV lamp and spray with *p*-anisaldehyde/ H_2_SO_4_ followed by heating.

#### Benzyl 4-hydroxy-3,5-dimethoxybenzoate (2) and benzyl 4-(benzyloxy)-3,5 dimethoxybenzoate (3)

A solution of syringic acid (320 mg, 1.6 mmol) and benzylbromide (1000 mg, 5.8 mmol) in *N,N*-dimethyl formamide (20 mL) was heated under reflux. Sodium hydride (38.4 mg, 1.6 mmol) was added portion-wise to the reaction mixture. The mixture was kept under reflux for 2 h. Reaction progress was monitored (thin layer chromatography, solvent system: 100% chloroform) and was shown go almost to completion. A saturated solution of sodium carbonate (40 mL) was added to the reaction mixture and, then, was extracted with chloroform (40 mL × 3). The combined chloroform layer was dried over anhydrous MgSO_4_, and evaporated *in vacuo* to afford a yellowish syrupy residue (1.06 g). This residue was chromatographed over flash silica gel column (106 g) using chloroform as the eluting solvent. This process afforded pure derivatives **2** (12 mg) and **3** (8 mg) as colourless oils. Spectral analysis confirmed the identity of **2** as benzyl 4-hydroxy-3,5-dimethoxybenzoate and that of **3** as benzyl 4-(benzyloxy)-3,5-dimethoxybenzoate. This reaction and chromatographic processes were scaled up and repeated several times to afford quantities enough to evaluate their biological activities (189 mg of **2**, and 124 mg of **3**).

Derivative **2**: yield, 2.6%; IR (neat, cm^-1^) ν _max_ 3345 (O-H), 1725 (C = O); ^1^H NMR (CDCl_3_, 600 MHz) see Table 2, supplemental data; ^13^C NMR (CDCl_3_, 125 MHz) see Table 2, supplemental data; High resolution ESIMS *m*/*z* 288.0942 [M]^+^ (calcd for C_16_H_16_O_5_ 288.0998).

**Table 2:** ^**1**^**H and **^**13**^**C NMR assignments for syringic acid and its derivatives 2, 5 and 6**^**a**^

**Position**	**Syringic acid**		**2**		**5**		**6**	
	**δ**_**C**_^***b***^	**δ**_**H **_**(m, *J *Hz)**	**δ**_**C**_^***b***^	**δ**_**H **_**(m, *J *Hz)**	**δ**_**C**_^***c***^	**δ**_**H **_**(m, *J *Hz)**	**δ**_**C**_^***b***^	**δ**_**H **_**(m, *J *Hz)**
1	128.6, C	-	121.1, C	-	121.1, C	-	121.0, C	-
2	107.0, CH	7.31 (s)	106.8, CH	7.36 (s)	106.9, CH	7.35 (s)	106.8, CH	7.38 (s)
3	148.0, C	-	146.7,C	-	146.7, C	-	146.6, C	-
4	142.6, C	-	139.4, C	-	139.4, C	-	139.4, C	-
5	148.0, C	-	146.7, C	-	146.7, C	-	146.6, C	-
6	107.0, CH	7.31 (s)	106.8, CH	7.36 (s)	106.9, CH	7.35 (s)	106.8, CH	7.38 (s)
1*'*	-	-	136.3, C	-	137.9, C	-	136.5, C	-
2*'*	-	-	128.2, CH	7.45 (dd, 7.8, 1.2)	113.8^*d*^, CH	6.98 (d, 2.2)	105.9, CH	6.59 (d, 2.4)
3*'*	-	-	128.6, CH	7.39 (dd, 7.8, 7.2)	160.0, C	-	160.9, C	-
4*'*	-	-	128.2, CH	7.34 (dd, 7.2, 1.2)	113.7^*d*^, CH	6.88 (dd, 8.1, 2.2)	100.0, CH	6.45 (dd, 2.4, 2.4)
5*'*	-	-	128.6, CH	7.39 (dd, 7.8, 7.2)	129.8, CH	7.30 (dd, 10.0, 8.8)	160.9, C	-
6*'*	-	-	128.2, CH	7.45 (dd, 8.8, 1.2)	120.4, CH	7.02 (d, 7.7)	105.9, CH	6.59 (d, 2.4)
7*'*-CH_2_	-	-	66.7, CH_2_	5.36 (s)	66.8, CH_2_	5.32 (s)	66.5, CH_2_	5.30 (s)
3,5-OCH_3_	56.1, CH_3_	3.86 (s)	56.5, CH_3_	3.93 (s)	56.6, CH_3_	3.93 (s)	56.5, CH_3_	3.95 (s)
3*'*-OCH_3_	-	-	-	-	55.3, CH_3_	3.82 (s)	55.4, CH_3_	3.82 (s)
5*'*-OCH_3_	-	-	-	-	-	-	55.4, CH_3_	3.82 (s)
OH	-	4.93 (s)	-	5.92 (s)	-	5.91 (br s)	-	5.95 (s)

^*a*^Spectra recorded in CDCl_3_. ^*b*^Multiplicities were determined by DEPT 135. ^*c*^Multiplicities were determined by APT. ^*d*^Assignments may be interchanged.

Derivative **3**: yield, 1.3%; IR (neat, cm^-1^) ν _max_ 1727 (C = O); ^1^H NMR (CDCl_3_, 600 MHz) see Table 3, supplemental data; ^13^C NMR (CDCl_3_, 125 MHz) see Table 3, supple-mental data; High resolution ESIMS *m*/*z* 378.1421 [M]^+^ (calcd for C_23_H_22_O_5_ 378.1467).

**Table 3:** ^**1**^**H and **^**13**^**C NMR assignments for syrinigc acid derivatives 3 and 4**^**a**^

**Position**	**3**		**4**	
	**δ**_**C**_^***b***^	**δ**_**H **_**(m, *J *Hz)**	**δ**_**C**_^***b***^	**δ**_**H **_**(m, *J *Hz)**
1	125.3, C	-	125.2, C	-
2	106.9, CH	7.31 (s)	107.0, CH	7.31 (s)
3	153.3, C	-	153.2, C	-
4	141.1, C	-	141.2, C	-
5	153.3, C	-	153.2, C	-
6	106.9, CH	7.31 (s)	107.0, CH	7.31 (s)
1*'*	136.1, C	-	137.6, C	-
2*'*	128.2, CH	7.44 (d, 7.8)	113.8, CH	7.44 (d, 7.8)
3*'*	128.5, CH	7.39 (dd, 7.8, 7.2)	159.8, C	7.39 (dd, 7.8, 7.2)
4*'*	128.0, CH	7.28 (dd, 7.2, 7.2)	113.6, CH	7.28 (dd, 7.2, 7.2)
5*'*	128.5, CH	7.39 (dd, 7.8, 7.2)	129.7, CH	7.39 (dd, 7.8, 7.2)
6*'*	128.2, CH	7.44 (d, 7.8)	120.4, CH	7.44 (d, 7.8)
1*'*	137.4, C	-	138.9, C	-
2*'*	128.2, CH	7.46 (d, 7.2)	113.5, CH	7.46 (d, 7.2)
3*'*	128.6, CH	7.34 (dd, 7.8, 7.2)	159.6, C	7.34 (dd, 7.8, 7.2)
4*'*	128.3, CH	7.32 (m)	113.9, CH	7.32 (m)
5*'*	128.6, CH	7.34 (dd, 7.8, 7.2)	129.2, CH	7.34 (dd, 7.8, 7.2)
6*'*	128.2, CH	7.46 (d, 7.2)	120.5, CH	7.46 (d, 7.2)
7*'*-CH_2_	66.8, CH_2_	5.36 (s)	66.7, CH_2_	5.36 (s)
7*'*-CH_2_	74.9, CH_2_	5.08 (s)	74.9, CH_2_	5.08 (s)
3,5-OCH_3_	56.3, CH_3_	3.86 (s)	56.3, CH_3_	3.86 (s)
3*'*-OCH_3_	-	-	55.2^*c*^, CH_3_	
5*'*-OCH_3_	-	-	-	-
3*'*- OCH_3_	-	-	55.3^*c*^, CH_3_	-
-COO-	166.2, C	-	166.2, C	-
OH	-	-	-	-

^*a*^Spectra recorded in CDCl_3_. ^*b*^Multiplicities were determined by DEPT 135. ^*c*^Assignments may be interchanged.

#### 3′-Methoxybenzyl 3,5-dimethoxy-4-(3′-methoxy benzyloxy)benzoate (4) and 3′-methoxybenzyl 4-hydroxy-3,5-dimethoxybenzoate (5)

Likewise, these derivatives were synthesized as mentioned above; however, 3-methoxybenzylbromide (920 mg, 4.6 mmol) was used, instead. Removal of un-reacted syringic acid was achieved *via* adding saturated solution of sodium carbonate and extraction with chloroform. Evaporation of chloroform layer yielded 1.03 g of a yellowish syrupy residue. This residue gave, after purification, pure derivatives **4** (10.6 mg) and **5** (15 mg) as pale yellow oils. Derivatives **4** and **5** identities were deduced from their spectral data. The reaction and purification processes were repeated to yield 93 mg of **4** and 131 mg of **5**.

Derivative **4**: yield, 1.5%; IR (neat, cm^-1^) ν _max_ 1727 (C = O); ^1^H NMR (CDCl_3_, 600 MHz) see Table 3, supplemental data; ^13^C NMR (CDCl_3_, 125 MHz) see Table 3, supplemental data; High resolution ESIMS *m*/*z* 438.1648 [M]^+^ (calcd for C_25_H_26_O_7_ 438.1679).

Derivative **5**: yield, 3%; IR (neat, cm^-1^) ν _max_ 3340 (O-H), 1727 (C = O); ^1^H NMR (CDCl_3_, 600 MHz) see Table 2, supplemental data; ^13^C NMR (CDCl_3_, 125 MHz) see Table 2, supplemental data; High resolution ESIMS *m*/*z* 318.1110 [M]^+^ (calcd for C_17_H_18_O_6_ 318.1103).

#### 3′,5′-dimethoxybenzyl 4-hydroxy-3,5-dimethoxy benzoate (6)

Following the above procedure, 3,5-dimethoxybenzylbromide ( 320 mg, 1.7 mmol) was used. This reaction was sluggish and never went to completion. Reaction workup, afforded 0.166 g of a yellowish syrupy residue which upon purification gave 5.4 mg of **6**. Derivative **6** identity was confirmed from spectral analysis to be 3′,5′-dimethoxybenzyl 4-hydroxy-3,5-dimethoxybenzoate. Reaction scale up afforded 52 mg of pure **6**.

Derivative **6**: yield, 1%; IR (neat, cm^-1^) ν _max_ 3340 (O-H), 1721 (C = O); ^1^H NMR (CDCl_3_, 600 MHz) see Table 2, supplemental data; ^13^C NMR (CDCl_3_, 125 MHz) see Table 2, supplemental data; High resolution ESIMS *m*/*z* 348.1200 [M]^+^ (calcd for C_18_H_20_O_7_ 348.1209).

### Biological activity

#### Cell Culture

All cell lines were obtained from ATCC (American Type Culture, VA, USA). Human colorectal cancer cell lines (CCL233 and CCL235) and Human breast cancer cell lines (HTB131 and HTB132) were cultivated in Leibovitz’s L15 medium, 90%, fetal bovine serum, 10%. L15-medium formulation is devised for use in a free gas exchange with atmospheric air. Human melanoma cell lines (HTB-66 and HTB-68) were cultivated in minimum essential medium Eagle with 2 mM L-glutamine and Earle’s BSS adjusted to contain 1.5 g/L sodium bicarbonate, 0.1 mM non-essential amino acids, 0.1 mM sodium pyruvate and Earl’s BSS, 90%, foetal bovine serum, 10%.

Normal human fibroblast cells (CCL1554) were cultivated in Eagle modified essential medium (90%) and foetal bovine serum, 10%.

#### Dose-dependent anti-mitogenic effect of syringic acid derivatives

The antimitogenic effects of syringic acid derivatives **2**–**6** toward panel of different human cancer cell lines comprised of colorectal (CCL233, CCL235), breast (HTB26, HTB132), breast (HTB26, HTB132), and melanoma (HTB66 and HTB68) cancer cell lines as well as normal human fibroblast CRL1554 cells were tested as previously described [31]. Human cancer cell lines and normal human fibroblast cells were plated in 96-well microtiter plates at a cell density of 27x10^3^cells/well. Cells were incubated in culture medium containing increasing concentrations of the tested derivatives (0.1, 0.4, 0.8, 1.2, 1.6 and 2.0 mg/mL) at 37°C in CO_2_ or non CO_2_ incubator depending on the cell lines for 144 h. On the completion of the treatment period, the media were discarded and 100 μl/well of MTT (5 mg/mL in culture medium filtered sterilized) was then added and the plate was incubated for 4 h at 37°C. The MTT solution was then aspirated and the formazan crystals were dissolved in 200 μl/well of 1:1 (v/v) solution of DMSO: ethanol for 20 min at ambient temperature. Change in absorbance was determined at A540 and 650 nm. Derivatives **2** (50–400 μg/mL), **5** (100–800 μg/mL) and **6** (260–400 μg/mL) were retested for their antimitogenic activities against human malignant melanoma cancer cell lines HTB66 and HTB68 and normal human fibroblast CRL1554 after 24 h of treatment as mentioned above.

#### Cell extract preparation

A whole-cell extract was prepared as previously described [7]. Briefly, human melanoma Cancer cells HTB68 were grown to 60-70% confluency, harvested, washed twice with PBS and homogenized in a lysis buffer (50 mM Tris (pH 8.0), 5 mM ethylenediaminetetraacetic acids, 150 mM NaCl, 0.5% NP40). After 30 minutes of rocking at 4°C, the mixtures were centrifuged at 14,000× g for 30 minutes and the supernatants were collected as whole-cell extracts.

#### Inhibition of the proteasome activities in human melanoma whole cell extracts by derivatives 2, 5 and 6

Various proteasomal activities were determined in human melanoma whole cell extract as previously described [31]. Briefly, human melanoma cancer cell extract (6 μg) was incubated for 90 min at 37°C with 20 μM fluorogenic peptide substrates: Suc-Leu-Leu-Val-Tyr-AMC (for proteasomal chymotrypsin-like activity), benzyloxycarbonyl(*Z*)-Leu-Leu-Glu-AMC (for proteasomal PGPH activity) and Z-Gly-Arg-AMC (for proteasomal trypsin-like activity) in 100 μl of the assay buffer in the presence or absence of Derivatives **2** (1.5 mg/mL), **5** (2.6 mg/mL) and **6** (1.75 mg/mL). After incubation, the reaction mixture was diluted to 200 μL with the assay buffer followed by a measurement of the hydrolysed 7-amido-4-methyl-coumarin (AMC) groups using a VersaFluor™ Fluorometer with an excitation filter of 380 nm and emission filter of 460 nm.

#### Flow cytometric analysis of cell cycle

The distribution of cells in cell cycle phases (Go/G1, S, G2/M) was determined using flow cytometry by the measurement of the DNA content of nuclei labelled with propidium iodide as previously described [51]. Briefly, human melanoma cell lines HTB66 and HTB68 were plated (2.5×10^5^ cells/mL) into 24-well plates and incubated at 37°C in CO_2_ incubator. Cells were treated with derivatives **2** (1.3 mg/mL) and **5** (1.9 mg/mL) for 24 h, starting 18 h after seeding the cells in culture. Untreated and derivative **5**-treated human melanoma cells were collected by trypsinization and then washed with cold phosphate buffered saline (PBS) and then counted. Cells were processed using DNA-prep kit (Beckman and Coulter, FL, USA) and a DNA-Prep EPICS workstation (Beckman and Coulter). During this process, cells were treated with a cell-membrane permeabilizing agent and then with propidium iodide (PI) and RNAase. The sample was then incubated at room temperature for 15 minutes before analysing by aligned flow cytometry (FC500, Beckman and Coulter). The percentage of cells in different cell cycle phases was calculated using the Phoenix statistical software package and Advanced DNA cell cycle software (Phoenix Flow System, San Diego, CA, USA).

#### Assessment of apoptosis by Annexin V-FITC and PI staining

The potential of derivatives **2** and **5** to induce apoptosis in human melanoma cells was determined by Annexin V-FITC and PI staining (Annexin V-FITC, BD Pharmingen, San Diego, CA, USA) and according to the manufacturer’s instruction. Briefly, human melanoma cell lines HTB66 and HTB68 were plated (2.5 × 10^5^ cells/mL) into 24-well plate and incubated at 37°C in CO_2_ incubator. Cells were treated with derivatives **2** (1.3 mg/mL) and **5** (1.9 mg/mL) for 24 h. Cells from control and treatment groups were re-suspended in 100 μl staining solution containing V fluorescein and propidium iodide in HEPES buffer. Following incubation at room temperature for 15 min, cells were analysed by flow cytometry. Annexin V binds to those cells that express phosphatidylserine on the outer layer of the cell membrane, and propidium iodide stains the cellular DNA of those cells with a compromised cell membrane. This allows for the discrimination of live cells (unstained with either fluorochrome) from apoptotic cells (stained only with V) and necrotic cells (stained with both V and propidium iodide).

### Molecular modelling studies

Three-dimensional structure building and all modelling were performed using the SYBYL Program Package, version X [52], installed on a DELL desktop workstation equipped with a dual 2.0 GHz Intel® Xeon® processor running the Red Hat Enterprise Linux (version 5) operating system. Conformations of bortezomib and syringic acid derivatives **2**–**6** were generated using Confort™ conformational analysis. Energy minimizations were performed using the Tripos force field with a distance-dependent dielectric and the Powell conjugate gradient algorithm with a convergence criterion of 0.01 kcal/(mol A). Partial atomic charges were calculated using the semiempirical program MOPAC 6.0 and applying the AM1.

Surflex-Dock Program version 2.0 interfaced with SYBYL-X was used to dock TMC-95A, bortezomib and syringic acid derivatives **2**–**6** in the active site of 20*S* yeast proteasome (PDB code: 2 F16 and 1JD2). Surflex-Dock employs an idealized active site ligand (protomol) as a target to generate putative poses of molecules or molecular fragments [53]. These putative poses were scored using the Hammerhead scoring function [53,54]. The 3D structures (PDB: 2 F16 and 1JD2) were taken from the Research Collaboratory for Structural Bioinformatics Protein Data Bank (http://www.rcsb.org/pdb).

## Competing interests

The authors declare that they do not have any financial or personal relationships with other people or organization that could inappropriately influence the work described in this manuscript.

## Authors’ contributions

KO and MA conceived of the study and designed it. KO and RG conducted chemical synthesis, isolation and identification of the derivatives. MA and RG performed all the biological activities evaluation. KE and AE conducted the molecular modeling studies. RA performed the flow cytometry. KO managed the funding acquisition form the funding agent, supervised the project and was, along with MA, involved in interpretation of data and compiled the manuscript. All authors have contributed and approved the final manuscript.
